# *Schinus terebinthifolius* Leaf Extract Causes Midgut Damage, Interfering with Survival and Development of *Aedes aegypti* Larvae

**DOI:** 10.1371/journal.pone.0126612

**Published:** 2015-05-14

**Authors:** Thamara Figueiredo Procópio, Kenner Morais Fernandes, Emmanuel Viana Pontual, Rafael Matos Ximenes, Aline Rafaella Cardoso de Oliveira, Carolina de Santana Souza, Ana Maria Mendonça de Albuquerque Melo, Daniela Maria do Amaral Ferraz Navarro, Patrícia Maria Guedes Paiva, Gustavo Ferreira Martins, Thiago Henrique Napoleão

**Affiliations:** 1 Departamento de Bioquímica, Centro de Ciências Biológicas, Universidade Federal de Pernambuco, Cidade Universitária, 50670–420, Recife, Pernambuco, Brazil; 2 Departamento de Biologia Geral, Universidade Federal de Viçosa, 36570–900, Viçosa, Minas Gerais, Brazil; 3 Departamento de Morfologia e Fisiologia Animal, Universidade Federal Rural de Pernambuco, Dois Irmãos, 52171–900, Recife, Pernambuco, Brazil; 4 Departamento de Antibióticos, Universidade Federal de Pernambuco, 50670–420, Recife, Pernambuco, Brazil; 5 Departamento de Biofísica e Radiobiologia-CCB, Universidade Federal de Pernambuco, Cidade Universitária, 50670–420, Recife, Pernambuco, Brazil; 6 Departamento de Química Fundamental, Centro de Ciências Exatas e da Natureza, Universidade Federal de Pernambuco, Cidade Universitária, 50740–560, Recife, Pernambuco, Brazil; Instituto de Biotecnología, Universidad Nacional Autónoma de México, MEXICO

## Abstract

In this study, a leaf extract from *Schinus terebinthifolius* was evaluated for effects on survival, development, and midgut of *A*. *aegypti* fourth instar larvae (L4), as well as for toxic effect on *Artemia salina*. Leaf extract was obtained using 0.15 M NaCl and evaluated for phytochemical composition and lectin activity. Early L4 larvae were incubated with the extract (0.3–1.35%, w/v) for 8 days, in presence or absence of food. Polymeric proanthocyanidins, hydrolysable tannins, heterosid and aglycone flavonoids, cinnamic acid derivatives, traces of steroids, and lectin activity were detected in the extract, which killed the larvae at an LC_50_ of 0.62% (unfed larvae) and 1.03% (fed larvae). Further, the larvae incubated with the extract reacted by eliminating the gut content. No larvae reached the pupal stage in treatments at concentrations between 0.5% and 1.35%, while in the control (fed larvae), 61.7% of individuals emerged as adults. The extract (1.0%) promoted intense disorganization of larval midgut epithelium, including deformation and hypertrophy of cells, disruption of microvilli, and vacuolization of cytoplasms, affecting digestive, enteroendocrine, regenerative, and proliferating cells. In addition, cells with fragmented DNA were observed. Separation of extract components by solid phase extraction revealed that cinnamic acid derivatives and flavonoids are involved in larvicidal effect of the extract, being the first most efficient in a short time after larvae treatment. The lectin present in the extract was isolated, but did not show deleterious effects on larvae. The extract and cinnamic acid derivatives were toxic to *A*. *salina* nauplii, while the flavonoids showed low toxicity. *S*. *terebinthifolius* leaf extract caused damage to the midgut of *A*. *aegypti* larvae, interfering with survival and development. The larvicidal effect of the extract can be attributed to cinnamic acid derivatives and flavonoids. The data obtained using *A*. *salina* indicates that caution should be used when employing this extract as a larvicidal agent.

## Introduction

The mosquito *Aedes aegypti* is the main vector of dengue, chikungunya, and yellow fever viruses. Vaccine is only available for yellow fever, and dengue can assume potentially lethal forms. About 2.3 million cases of dengue were reported in the Americas in 2013, and thus, dengue was ranked among the most important viruses transmitted by arthropods [1], [2]. Prophylactic methods mainly focus on the control of vector population with insecticides, improvement of sanitation, and strengthening community efforts in order to eliminate breeding sites [3].

Currently, chemical control of *A*. *aegypti* has faced a number of difficulties such as environmental persistence and unselective toxicity of the commonly used insecticides; further, the emergence of resistant populations has been reported [4], [5], [6], [7], [8]. A reasonable alternative is to search for natural insecticides for the control of mosquito populations since they are usually highly biodegradable, and the availability of more alternatives broadens the range for regular interchange and alternation of insecticides, minimizing resistance development [9], [10].

Plants produce many defensive compounds, which have been widely evaluated as insecticides. Plant extracts and essential oils contain several types of secondary metabolites that exert deleterious effects on insects. Proteins such as protease inhibitors and lectins have been also reported to act as insecticidal agents. In addition to causing insect mortality at all life stages, plant-derived insecticides may also disrupt metamorphosis, promote morphological alterations, and exert irritant and repellent effects [11], [12], [13], [14], [15], [16].

Many plant insecticides primarily target the midgut of the mosquito larvae, and some are able to interfere with the larval development into adult stage, even at sub-lethal concentrations [17]. The metamorphosis of *A*. *aegypti* larvae comprises comprehensive transformations of the insect body, including a remodeling of the midgut, where larval digestive cells are completely replaced [18], [19], [20]. In this sense, deleterious effects of plant compounds on the midgut may interfere with larval development.

*Schinus terebinthifolius* Raddi (Brazilian pepper tree) is a plant from the Anacardiaceae family native to Central and South America, and found in Brazil from the northeast to the south, in plant communities such as the Atlantic Forest and the Cerrado [21]. It is a source of many bioactive compounds, and its leaves are popularly used in remedies for healing ulcers and wounds, combating oral candidiasis in children, and for producing infusions considered to have anti-rheumatic properties [22], [23]. Some of the compounds from *S*. *terebinthifolius* leaves with proven biological activities are terpenes that induce melanoma apoptosis and protect against metastasis [24], [25], essential oil that inhibits mitosis in lettuce and onion [26], aromatic compounds with the ability to treat allergies [27], and a lectin (called *S*. *terebinthifolius* leaf lectin, SteLL) with antimicrobial properties [28]. Larvicidal activity against *A*. *aegypti* has been detected in a dichloromethane extract from *S*. *terebinthifolius* leaves and an essential oil extracted from its fruit [29], [30].

This study shows the effects of a saline extract from *S*. *terebinthifolius* leaves on the survival, development, and midgut of *A*. *aegypti* fourth instar larvae (L_4_). The extract was also evaluated for phytochemical composition and toxicity to *Artemia salina*. Solid phase extraction of the main secondary metabolites detected in the extract separated them into three fractions. In addition, the lectin SteLL was isolated from the extract. The fractions and the lectin were then evaluated for deleterious effects on larvae.

## Materials and Methods

### Plant material

The leaves of *S*. *terebinthifolius* were collected in the campus of the *Universidade Federal de Pernambuco* at Recife, Brazil, and left to dry at 28°C during 3–4 days. Next, the leaves were powdered using a blender and stored at -20°C. A voucher specimen is archived under number 73,431 at the herbarium from the *Instituto Agronômico de Pernambuco* (IPA), Recife, Brazil. Plant collection was performed with authorization (number 36301–2) of the *Instituto Chico Mendes de Conservação da Biodiversidade* (ICMBio) from the Brazilian Ministry of Environment.

### *Aedes aegypti* larvae

Larvae were reared in insectaries from the *Laboratório de Ecologia Química* (LEQ) of the *Universidade Federal de Pernambuco* and from the *Departamento de Biologia Geral* (DBG) of the *Universidade Federal de Viçosa*. The colonies belong to the Rockefeller (LEQ) and PPCampos (DBG) strains, respectively. Rockefeller and PPCampos larvae were hatched in dechlorinated water containing cat food (Whiskas) and the colonies were reared at a 26 ± 1°C, relative humidity 75 ± 10%, and photoperiod 12L:12D. The larvae were collected for use in the bioassays when they reached the early fourth instar (L_4_) stage.

### *Schinus terebinthifolius* leaf extract

The extract preparation was started by homogenizing 10 g of leaf powder with 100 mL of 0.15 M NaCl during 16 h at 28°C, using a magnetic stirrer. Next, the suspension was filtered through filter paper and centrifuged (3,000 *g*, 15 min) and dialyzed (4 h) against distilled water. The dialyzed supernatant, corresponding to the leaf extract, was then lyophilized to dryness for 24 h using a freeze-dryer (LIOTOP L101, Liobras, São Carlos, Brazil) at a temperature of -45°C and a vacuum of 300 μmHg below atmospheric pressure. After lyophilization, the leaf extract was ressuspended in 0.15 M NaCl to a concentration of 2.7% (dry weight/volume).

### Phytochemical analysis, hemagglutinating activity and evaluation of microorganism presence in leaf extract

The phytochemical screening of the extract was performed by thin-layer chromatography (TLC) on silica plates (60F254, aluminum backed, 200 μm layer thickness, 8.0 x 5.0 cm, Merck, Darmstadt, Germany). The presence of alkaloids, triterpenes, steroids, cinnamic acid derivatives, heterosid and aglycone flavonoids, hydrolysable tannins, and proanthocyanidins were investigated using the adequate development systems and revealers listed in Table 1 [31], [32], [33]. After development, the plates were air dried and sprayed with the revealers in a fume hood.

**Table 1:** Development systems and revealers used for analysis by thin-layer chromatography of secondary metabolites in *S*. *terebinthifolius* leaf extract.

Secondary metabolites	Development system	Revealer
**Alkaloids**	EtOAc/HCOOH/AcOH/H_2_O (100:11:11:26 v/v)	Dragendoff’s reagent
**Triterpenes and steroids**	EtOAc/HCOOH/AcOH/H_2_O (100:0.5:0.5:0.5 v/v)	Lieberman-Burchard’s reagent
**Aglycone and flavonoid heterosids**	EtOAc/HCOOH/AcOH/H_2_O (100:11:11:27 v/v)	Neu’s reagent
**Proanthocyanidins**	EtOAc/HCOOH/AcOH/H_2_O (100:11:11:26 v/v)	Vanilin-chloridric acid
**Cinnamic acid derivatives**	EtOAc/HCOOH/AcOH/H_2_O (100:11:11:27 v/v)	Neu’s reagent

The presence of lectin in leaf extract was investigated by determining the hemagglutinating activity in 96-well microtiter plates (TPP-Techno Plastic Products, Trasadingen, Switzerland). The assay was performed by a twofold serial dilution of extract (50 μL) in 0.15 M NaCl followed by addition to each well of 50 μL of a suspension (2.5% v/v) of glutaraldehyde-treated rabbit erythrocytes in 0.15 M NaCl. The plate was incubated at 27°C for 45 min. Hemagglutinating activity was quantified as the reciprocal value of the highest dilution of sample that promotes full agglutination of erythrocytes [34]. Specific activity was calculated by the ratio between the hemagglutinating activity and the protein concentration, which was determined according to Lowry, et al. [35].

In order to evaluate the presence of microorganisms in leaf extract, aliquots (100 μL) of the extract were smeared on petri dishes containing Mueller Hinton Agar, Sabouraud-Dextrose Agar or Potato-Dextrose Agar. Plates were incubated at 37°C for 24 h. After this period, the microbial growth was observed, and the number of colony forming units (CFU) was determined.

### Effects of leaf extract on survival and development of *A*. *aegypti* larvae

First, bioassays were performed without addition of food (unfed larvae), according to the method described by Navarro, et al. [36] and following the instructions of the World Health Organization [37]. The leaf extract was diluted with distilled water in order to obtain test solutions in the concentration range 0.3–1.35% (w/v). In each assay, 20 early L_4_ larvae (Rockefeller strain) were placed into disposable plastic cups containing 20 mL of the test solution or 0.15 M NaCl (control). The assays were maintained at 26 ± 1°C, relative humidity 75 ± 10%, and photoperiod 12L:12D. Two independent experiments were performed in triplicate. The number of live and dead larvae, pupae, and adults was counted daily until the 8^th^ day.

Next, the bioassays were performed with food supplied to larvae (fed larvae). At the beginning of the incubation period, 0.05 g of cat food (Whiskas) was added per cup. The tested concentrations and laboratory conditions were the same described above.

### Effects of leaf extract on the midgut of the larvae

#### Bioassays and fixation of midguts

Leaf extract was diluted with distilled water in order to obtain a test solution at 1.0% (w/v). Next, 20 early L_4_ larvae (PPCampos strain) were transferred to plastic vessels containing 20 mL of the test solution or 0.15 M NaCl (control). Food was added (0.05 g) in each vessel. The assays were maintained at 26 ± 1°C, relative humidity 75 ± 10%, and photoperiod 12L:12D. After 12 h, the midguts of ten larvae from each treatment were dissected in a physiologic solution for insects (0.1 M NaCl, 20 mM KH_2_PO_4_, 20 mM Na_2_HPO_4_). Some larvae and midguts were observed using a stereomicroscope and photographed using a digital camera. The dissected midguts were fixed in formaldehyde and picric acid solution (Zamboni’s solution), except those to be analyzed using a transmission electron microscope, which were fixed in 2.5% glutaraldehyde in 0.1 M sodium cacodylate (pH 7.2) for 2 h. Midguts obtained from the control larvae were photographed and fixed in a similar manner.

To assess the occurrence of melanization in the midgut of treated larvae, 20 L4 were exposed to the extract at 1.0% (w/v), containing the phenoloxidase inhibitor phenylthiourea (PTU) (0.01 M). A separate group of larvae was incubated only with PTU. Treated and control larvae received food as described above. After incubation for 12 h, the midguts were dissected, and observed under the stereomicroscope.

#### Histology analysis

Fixed midguts (of larvae from controls and treatment with the extract alone) were washed with distilled water, dehydrated in a graded series of ethanol (70–100%), and embedded in Historesin (Leica, Solms, Germany). Next, the material was cut into 3-μm sections, stained with toluidine blue, and mounted in Eukitt medium (Fluka, USA). The stained midguts were observed under an optical microscope (Olympus BX60, Olympus America, Inc., NY, USA) and photographed using a digital camera.

#### Transmission electron microscopy

Fixed midgut fragments were washed in cacodylate buffer and post-fixed in 1% osmium tetroxide for 2 h in the dark. Following post-fixation, the material was washed twice with 0.1 M phosphate-buffered saline (PBS), dehydrated in an increasing series of ethanol concentrations (70–100%), and pre-infiltrated in a LR white resin solution and 100% ethanol (2:1) for 1 h. The samples were then embedded in pure resin and maintained at 25°C for 16 h, followed by polymerization in gelatin capsules (Electron Microscopy Sciences) at 60°C for 24 h. Ultrathin sections were placed on copper grids and incubated for 20 min in 1% aqueous uranyl acetate and lead citrate. The samples were observed and photographed using a Zeiss EM 109 microscope (Carl Zeiss AG, Oberkochen, Germany).

#### Fluorescence microscopy

The nuclei of cells from the fixed midgut were stained with diamidino-2-phenylindole (DAPI; Biotium, USA) for 30 min. The midguts were mounted on slides using Mowiol antifading solution (Sigma-Aldrich, MO, USA) and analyzed in an epifluorescence microscope Olympus BX-60 coupled to the capture system Olympus Q-Color 3 (Olympus America, Inc., NY, USA). Six areas of each region (anterior and posterior) of the midgut were randomly photographed with a 40× objective lens (total area = 0.414 mm^2^) [38]. The number of digestive cells (larger nuclei and present at the apical region of digestive epithelium) and regenerative cells (small nuclei and found at the basal region of epithelium) in the midgut were counted as described elsewhere [19], [20].

Enteroendocrine cells were identified by labeling the peptide FMRFamide [20], which is usually abundant in endocrine cells of the digestive tract in insects. Fixed midguts were washed three times for 30 min with PBST (phosphate buffered saline with 0.05% Tween—Sigma-Aldrich, USA), and then incubated for 24 h at 4°C with a solution of anti-FMRFamide primary antibody (Peninsula Lab, UK) prepared (1:400) in 1% PBST. After washing with PBS three times (5 min each), the midguts were incubated with a secondary antibody conjugated with fluorescein isothiocyanate (FITC) (Sigma-Aldrich, USA) for 24 h at 4°C. The midguts were washed three times with PBS, mounted using Mowiol solution, and observed under the epifluorescence microscope for counting of enteroendocrine cells.

Proliferating cells in the midgut were investigated by *in situ* labeling of the mitosis marker phospho-histone H3 [39]. Fixed guts were incubated for 24 h at 4°C with the primary antibody anti-phospho-histone H3 (Cell Signaling, USA) prepared (1:100) in PBS with 1% Tween (PBST). Samples were washed three times with PBS and incubated for 24 h at 4°C with the FITC-conjugated secondary antibody (Sigma-Aldrich, USA) diluted (1:500) in PBS. After three 10-min washing steps with PBS, the slides were mounted using Mowiol solution, analyzed, and photographed in a Zeiss LSM 510 confocal microscope (Carl Zeiss AG, Oberkochen, Germany). Positive cells were counted along the entire midgut.

DNA fragmentation was identified *in situ* using the Cell Death Detection kit, Fluorescein (TUNEL reaction), from Roche (Basel, Sweden). Fixed midguts were treated with proteinase K (Sigma-Aldrich, USA) at 10–20 μg/mL, in 10 mM Tris-HCl, pH 7.4, for 60 min at 37°C. Next, they were washed with PBS and incubated for 60 min with TUNEL solution at 37°C. The slides were then mounted and analyzed as described above.

### Separation of secondary metabolites, lectin isolation and bioassays

Secondary metabolites in the leaf extract were semi-purified by solid phase extraction (SPE) on a 24-port vacuum manifold (Supelco, PA, USA). The SPE cartridges, Chromabond C18 (500 mg/3 mL) from Macherey-Nagel (Düren, Germany), were preconditioned with 1 mL of methanol and equilibrated with 3 mL of 0.1 M Tris-HCl pH 9.0. The extract was dissolved in Tris buffer to 25 mg/mL and filtered through a 0.45 μm syringe filter. Then, 1 mL of the extract solution was loaded into the cartridge, which was washed with Tris buffer for the elution of the cinnamic acid derivatives (fraction 1, F1). Flavonoids were then eluted with 2 mL of methanol (fraction 2, F2), and hydrolysable tannins were eluted with 2 mL of 1:1 (v/v) methanol-acetic acid (fraction 3, F3). F1 was dialyzed for removal of Tris molecules. After evaporation of the solvents, the fractions were once again submitted to phytochemical screening by TLC as described above.

The *S*. *terebinthifolius* leaf lectin (SteLL) was isolated from leaf extract according to the procedure described by Gomes, et al. [28]. The extract was loaded onto a chitin (Sigma-Aldrich, MO, USA) column (7.5×1.5 cm) equilibrated with 0.15 NaCl at a flow rate of 20 mL/h. The unadsorbed material was removed with equilibrating solution after absorbance at 280 nm was lower than 0.020. Next, SteLL was eluted from the column with 1.0 M acetic acid. The isolated lectin was then dialyzed in a 10-kDa cut-off membrane (Sigma-Aldrich, MO, USA) against distilled water (4 h) and evaluated for hemagglutinating activity and protein concentration.

Larvicidal assays with F1, F2 and F3 (1.0% w/v, dissolved in distilled water) and SteLL (0.05–1.0 mg/mL, in water) were performed as described above, with food addition. The number of live and dead larvae, pupae, and adults was counted daily until the 8^th^ day.

### Environmental toxicity assay using *Artemia salina*

*A*. *salina* eggs were acquired from local pet shops. The eggs were incubated at 27±2°C in natural seawater with pH adjusted to 8.0. After 24 h, the hatched nauplii were collected and used in bioassays. Groups of 12–15 larvae were exposed to 5-mL solutions of leaf extract (0.125–1.0%), F1 (1.0%, w/v) or F2 (1.0%, w/v) diluted in natural seawater and, after 24 h, the survival rates (%) were recorded [40]. In the control group, larvae were incubated in seawater. Three independent experiments were performed in triplicate.

### Statistical analysis

Standard deviations (SD) were calculated using GraphPad Prism version 4.0 for Windows (GraphPad Software, San Diego, California, USA), and the data were expressed as replicate means ± SD. The lethal concentrations required for killing 50% of *A*. *aegypti* larvae (LC_50_) after 3 and 8 days were calculated by probit analysis with a reliability interval of 95% using the StatPlus 2006 software (AnalystSoft, Canada). The results from midgut cell counting were submitted to variance analysis (ANOVA) when distribution was considered normal or to Kruskal-Wallis’s test in cases with non-normal distribution.

## Results

Phytochemical screening of leaf extract revealed the presence of polymeric proanthocyanidins, heterosids and aglycone flavonoids, hydrolysable tannins, and mainly cinnamic acid derivatives. Trace amounts of steroids were also detected, as well as lectin (specific hemagglutinating activity of 81). No microbial growth, including bacteria and yeasts, was observed in the leaf extract smeared on plates with the culture media.

**Fig 1:**
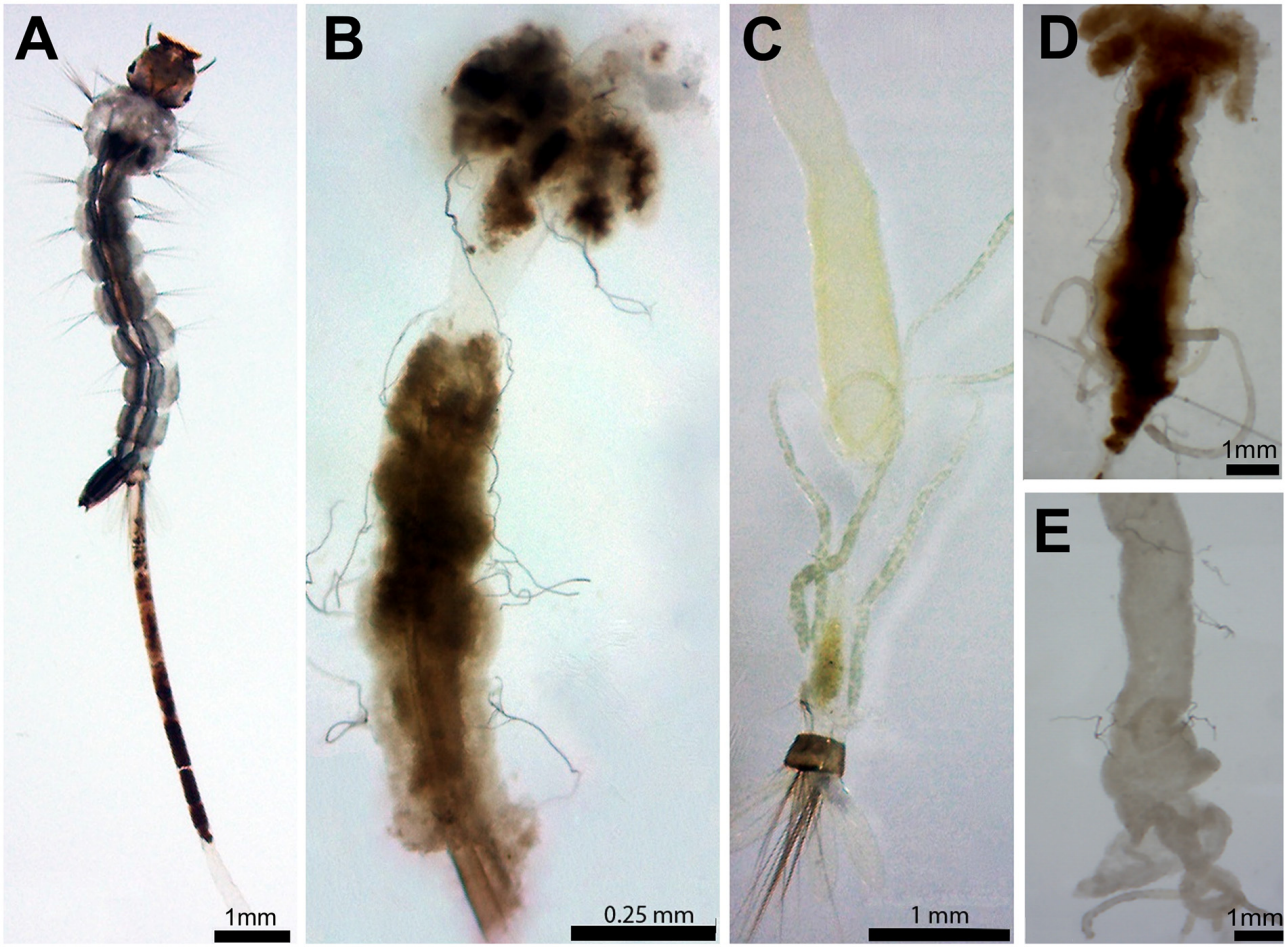
*Aedes aegypti* L_4_ larvae incubated for 12 h with *Schinus terebinthifolius* leaf extract (1.0%, w/v). (A) Larva eliminating the gut content covered by the peritrophic matrix. (B) Shrunken and pigmented midgut dissected from a larva incubated with the leaf extract. (C) Midgut dissected from a control larvae, after removal of gut content and peritrophic matrix, without apparent alterations. (D) Midgut dissected from a larva incubated with the leaf extract containing the 0.01 M phenylthiourea (PTU), a phenoloxidase inhibitor. (E) Midgut dissected from a larva incubated with 0.01 M PTU.

After 24 h, mortality rates of unfed larvae at concentrations from 0.5% to 1.35% ranged between 3.3% and 25%, while in concentrations below 0.5% and in the control there was no mortality (Table 2). Interestingly, in all treatments with extract, there were larvae that eliminated the gut content, which was enclosed in the peritrophic matrix (Fig 1A). All the larvae exposed to highest extract concentrations showed this reaction (Table 2).

**Table 2:** Acute effects of *Schinus terebinthifolius* leaf extract on *Aedes aegypti* larvae after 24 h, in bioassays performed adding or not food in the start of experiment.

Concentration (%)	Mortality rate (%)		Larvae that eliminated gut content (%)	
	Without food addition	With food addition	Without food addition	With food addition
**0.3**	0.0 a	0.0 a	5.0 ± 0.0 a	6.7 ± 2.8 a
**0.4**	0.0 a	0.0 a	10.0 ± 5.0 b	11.7 ± 2.8 b
**0.5**	3.3 ± 2.8 b	0.0 a #	15.0 ± 0.0 c	18.3 ± 2.8 c
**0.75**	8.3 ± 2.8 c	0.0 a #	40.0 ± 0.0 d	35.0 ± 0.0 d #
**1.2**	21.6 ± 2.8 d	5.3 ± 0.2 b #	100 e	100 e
**1.35**	25.0 ± 0.0 e	30.0 ± 0.0 c #	100 e	100 e
**Control**	0.0 a	0.0 a	0.0 f	0.0 f

Different letters at the same column indicate significant differences (p<0.05) between control and the treatments at different concentrations. The symbol # indicates significant difference (p<0.05) between the value in assay with food addition and that obtained in assay without food addition.

The LC_50_ values of 1.05% and 0.62% were determined for unfed larvae after 3 and 8 days, respectively, with no mortality in the control during these periods. At the 8^th^ day, the number of individuals at pupa stage was significantly higher (p < 0.05) in the control than in all other treatments while the number of emerged adults was similar to the control (p > 0.05) only in the treatments at concentrations of 0.3% and 0.4% (Table 3). In the bioassays at the concentrations of 1.2% and 1.35%, all the individuals died at the larval or pupal stages.

**Table 3:** Mortality and life stages reached after 8 days by *A*. *aegypti* individuals incubated with *S*. *terebinthifolius* from the fourth larval stage.

Treatment / Concentration	Life stage				
	Larvae (%)		Pupae (%)		Adults (%)
	Live	Dead	Live	Dead	
**Without food**					
**Control**	71.6 ± 2.8 a	0.0 a	16.7 ± 2.8 a	0.0 a	11.7 ± 2.8 a
**0.3%**	73.3 ± 2.8 a	10.3 ± 0.6 b	3.3 ± 2.8 b	0.0 a	13.0 ± 2.6 a
**0.4%**	66.7 ± 2.8 a	16.7 ± 2.8 c	3.3 ± 2.8 b	0.0 a	13.3 ± 2.8 a
**0.5%**	71.6 ± 2.8 a	20.0 ± 5.0 c	3.3 ± 2.8 b	0.0 a	5.0 ± 0.0 b
**0.75%**	13.3 ± 2.8 b	83.3 ± 2.8 d	0.0 c	0.0 a	3.3 ± 2.8 c
**1.2%**	0.0 c	98.3 ± 2.8 e	0.0 c	1.6 ± 2.8 b	0.0 d
**1.35%**	0.0 c	100 f	0.0 c	0.0 a	0.0d
**With food**					
**Control**	28.3 ± 2.8 a #	0.0 a	10.0 ± 0.0 a #	0.0 a	61.7 ± 2.8 a #
**0.3%**	70.0 ± 0.0 b	0.0 a #	5.0 ± 0.0 b	0.0 a	25.0 ± 0.0 b #
**0.4%**	85.0 ± 0.0 c #	10.0 ± 0.0 b #	0.0 c #	0.0 a	5.0 ± 0.0 c #
**0.5%**	73.3 ± 2.8 d	26.7 ± 2.8 c	0.0 c #	0.0 a	0.0 d #
**0.75%**	68.3 ± 2.8 bd #	31.6 ± 2.8 c #	0.0 c	0.0 a	0.0 d #
**1.2%**	58.3 ± 2.8 e #	41.6 ± 2.8 d #	0.0 c	0.0 a #	0.0 d
**1.35%**	0.0 f	98.3 ± 2.8 e	0.0 c	1.7 ± 2.8 b #	0.0 d

Different letters at the same column indicate significant differences (p<0.05) between control and the treatments at different concentrations. The symbol # indicates significant difference (p<0.05) between the value in assay with food addition and that obtained in assay without food addition.

For fed larvae, *S*. *terebinthifolius* leaf extract had somewhat distinct effects on mosquito survival. Table 2 shows that there was no mortality after 24 h using the extract at 0.3–0.75% concentrations, and the mortality rate at 1.2% was much lower than when food was unavailable. On the other hand, the number of dead larvae at the concentration of 1.35% was similar in bioassays with unfed and fed larvae. The estimated LC_50_ for 3 (1.3%) and 8 (1.03%) days were higher than those for bioassays without food. After 24 h, the fed and treated larvae also eliminated the contents of the gut, in numbers similar to those in assays of unfed larvae (Table 2).

The leaf extract clearly led to disruptions in development of *A*. *aegypti* in the assay with fed larvae (Table 3). By the 8^th^ day, most of the individuals (61.7%) had emerged as adults in the control, while in the treatments with extract at 0.3% and 0.4%, this number was 25.0 and 5.0%, respectively, and there was no emergence of adults in bioassays at any of the other concentrations.

Larvae from PPCampos strain were treated with the leaf extract at 1.0% (w/v) and the mortality rate was similar to that detected for Rockefeller larvae; also, the PPCampos larvae (100%) eliminated the gut content. Fig 1B shows a shrunken and dark midgut dissected from a fed larva incubated (12 h) with the extract (1.0%). The midgut darkening was still observed after larvae incubation with the leaf extract (1.0%) containing 0.01 M PTU (Fig 1D). The midgut of larvae incubated only with PTU (Fig 1E) was similar to that of control (Fig 1C) (i.e., without the extract and/or PTU).

**Fig 2:**
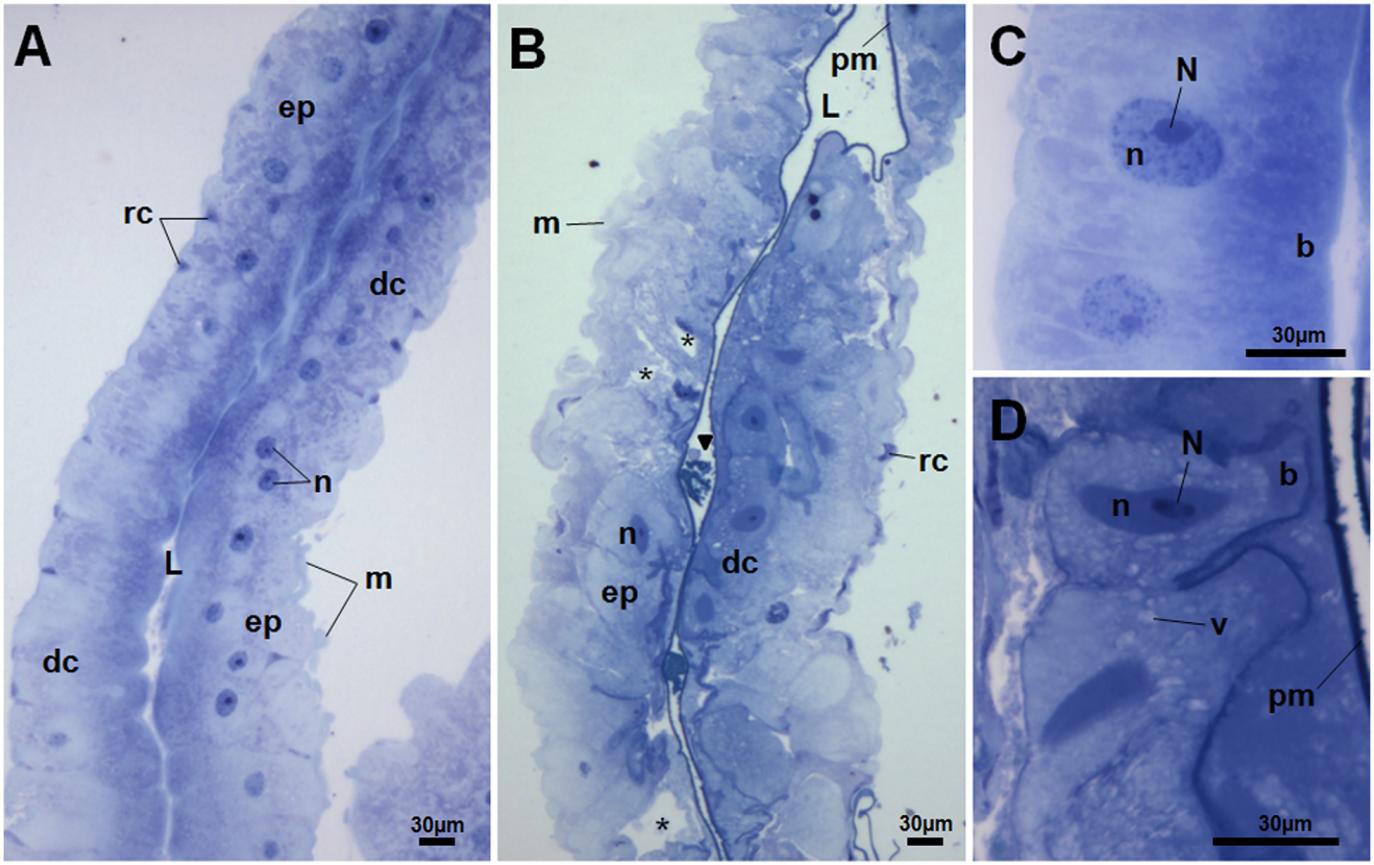
Toluidine Blue stained histological sections of the midgut of *Aedes aegypti* L4 from control (A) and incubated for 12 h with the *Schinus terebinthifolius* leaf extract (B). Midgut from control larva (A) showed a single-layered epithelium (ep) comprised of digestive (dc) and regenerative cells (rc) with preserved morphology (C). L, midgut lumen; m, muscle; n, digestive cell nuclei. Midgut from treated larva (B) showed intense disorganization of the epithelial layer (ep) with several spaces between cells (*) and some hypertrophied digestive cells (dc). Tissue/cell debris (arrowhead) is seen in the midgut lumen. m, muscle; n, digestive cell nucleus; pm, peritrophic matrix. Details of columnar digestive cells for control (C) and treated (D) larvae. Structure resembling vacuoles (v) are seen in D. n, cell nucleus; N, nucleolus; B, brush border.

**Fig 3:**
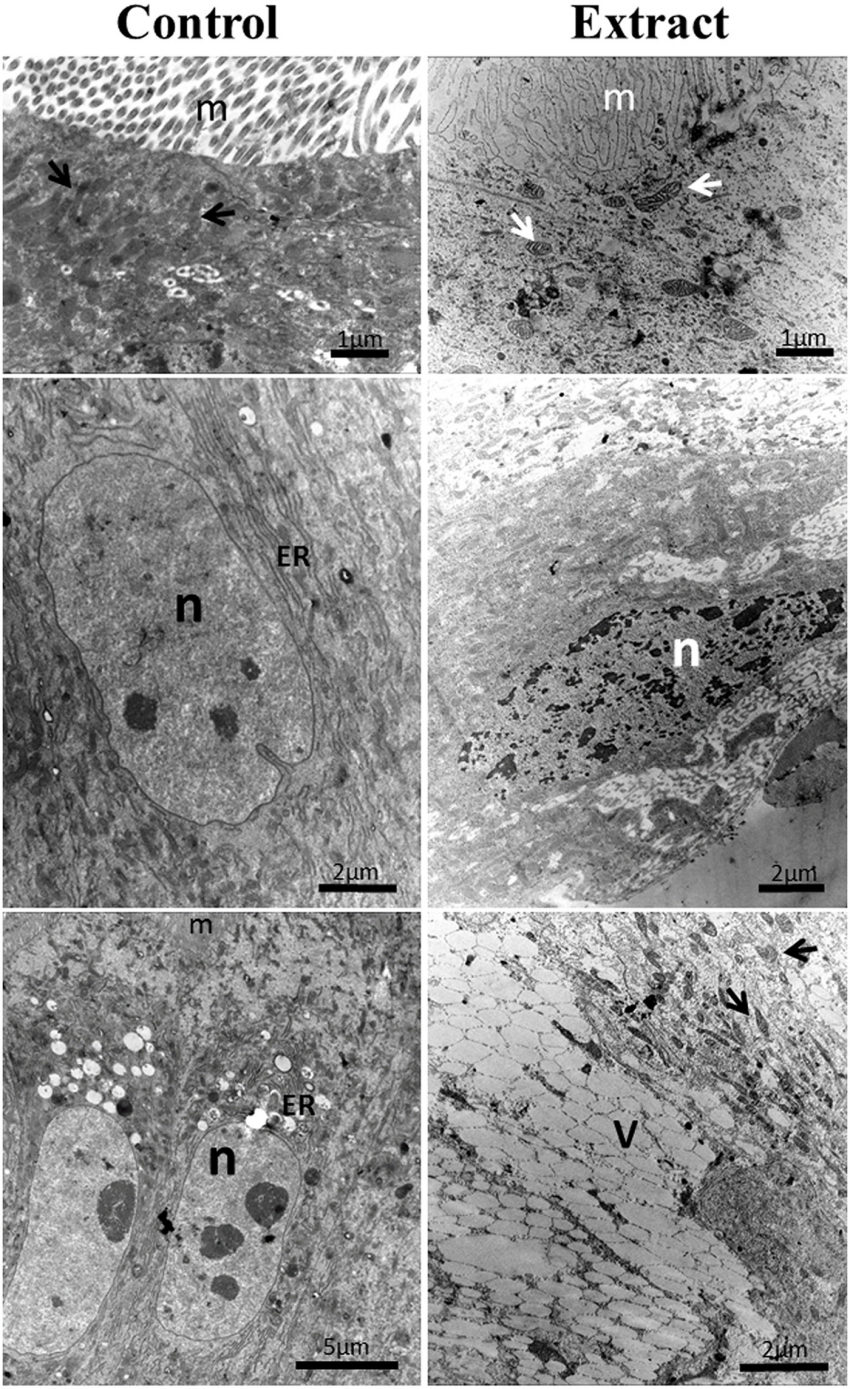
Electron micrographs of the midguts of *Aedes aegypti* L4 from control and those treated with *Schinus terebinthifolius* leaf extract at 1.0% (w/v). Mitochondria (arrowheads); microvilli (m); electron-lucent vacuoles (V); endoplasmic reticulum (ER); nucleus (n).

Histology analysis revealed that the midgut epithelium of fed larvae incubated with the extract had remarkable disorganization in comparison to the control, with several spaces between cells and the presence of tissue/cell debris in the luminal space (Fig 2A and 2B). A thin peritrophic matrix can still be seen in the midgut of exposed larvae. Deformations and hypertrophy of epithelial cells were also observed, as well as the presence of structures resembling vacuoles (Fig 2D). Ultrastructural analysis by transmission electron microscopy displayed drastic cell disruption in the midgut. Digestive cells from larvae treated with leaf extract showed disrupted microvilli, cytoplasm electron-lucent, and vacuolated cytoplasm (Fig 3).

**Fig 4:**
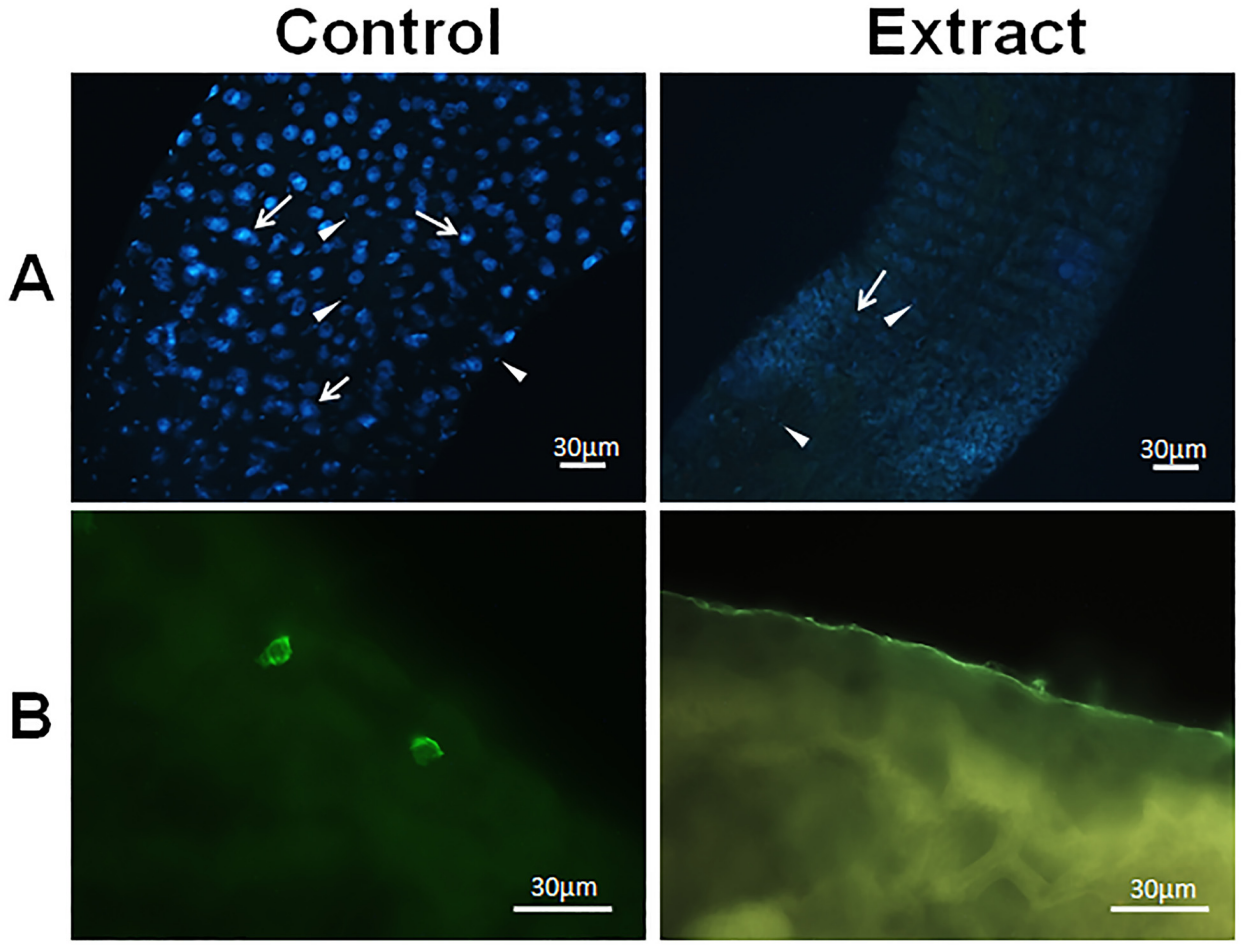
Midgut of *Aedes aegypti* L4 incubated for 12 h with distilled water (control) and *Schinus terebinthifolius* leaf extract at 1.0% (w/v). (A) Total mounting of larvae midgut stained with DAPI (blue) and displaying the nuclei of digestive (arrow) and regenerative (arrowhead) cells. (B) Staining of enteroendocrine (FMRF-imunorreactive) cells at the posterior region of midgut.

**Fig 5:**
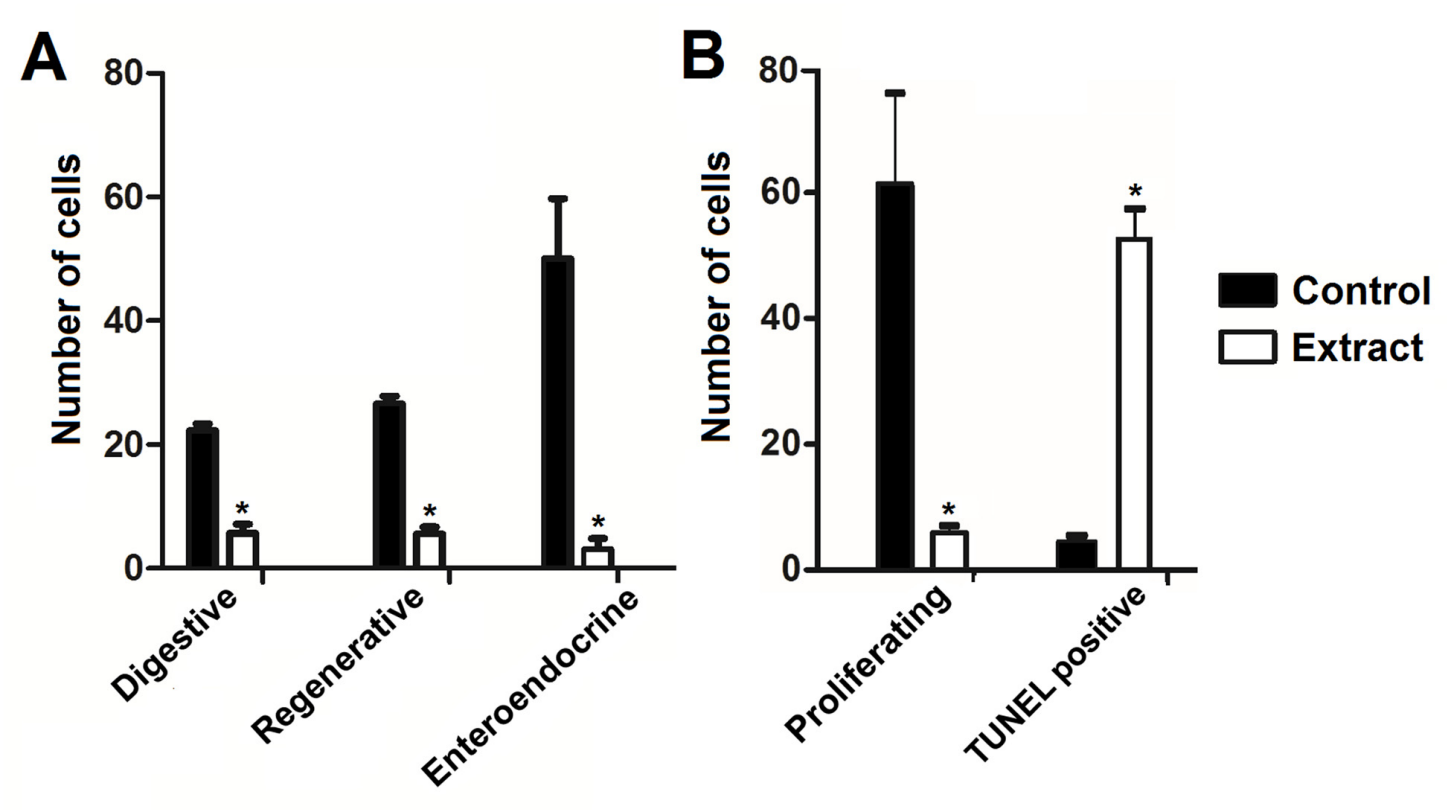
Number of different cell types in the midgut of *Aedes aegypti* L_4_ from control and those incubated for 12 h with the *Schinus terebinthifolius* leaf extract (1.0%, w/v). (A) Digestive, regenerative, and enteroendocrine cells from the midgut epithelium were counted under the fluorescence microscope. (B) Number of proliferating regenerative cells or cells with nuclear DNA damage (TUNEL positive) in the midgut epithelium were determined by fluorescence microscopy. (*) indicates significant difference (p < 0.05) in comparison to the control.

Cell nuclei from the midgut were intensely stained with DAPI in the control treatments, but only weakly stained nuclei could be seen in midgut of treated larvae (Fig 4A). In addition, enteroendocrine (FMRF-immunoreactive) cells were seen in the midgut of control larvae, but they were rarely seen in the midgut of treated individuals (Fig 4B). The numbers of digestive, regenerative, and enteroendocrine cells counted in the midgut of treated larvae were remarkably lower than that in the control (Fig 5A).

**Fig 6:**
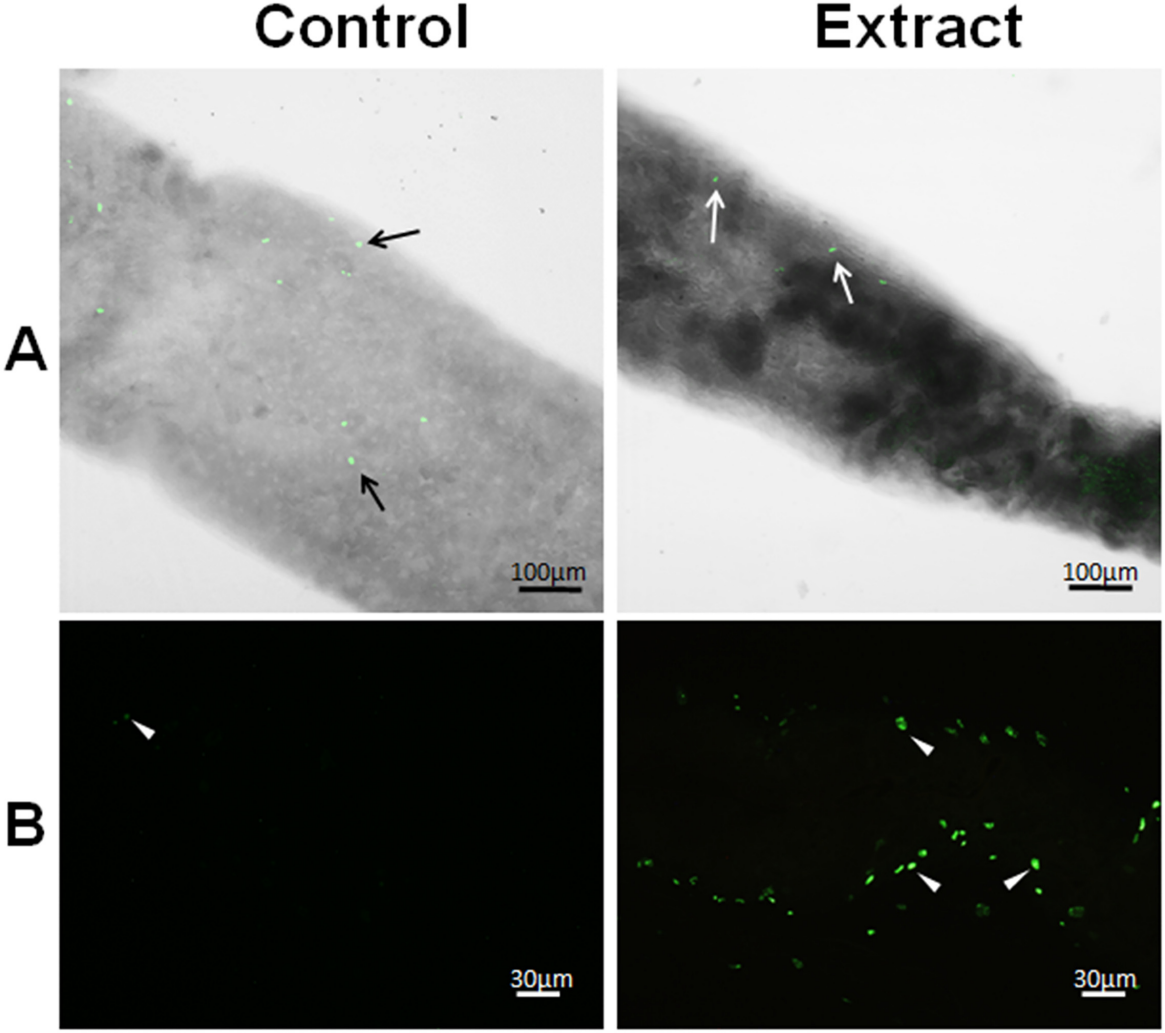
Proliferating regenerative cells or cells with nuclear DNA damage in the midgut of *Aedes aegypti* L_4_ incubated for 12 h with distilled water (control) and *Schinus terebinthifolius* leaf extract at 1.0% (w/v). (A) Nuclei of proliferating (phosphohistone H3-positive) regenerative cells (arrow) at the posterior region of the midgut. (B) Nuclei of digestive and regenerative cells positive for DNA damage/fragmentation (arrowhead).

Many proliferating cells (phosphohistone H3-positive) were seen in the midgut of the control larvae but they were scarce in the midgut of treated larvae (Figs 5B and 6A). In addition, DNA fragmentation was detected by the TUNEL reaction in the midgut of larvae exposed to the leaf extract at much higher levels than in the control (Figs 5B and 6B).

After the phytochemical screening, the leaf extract was semi-purified by SPE for separation of the main classes of secondary metabolites. The TLC revealed that F1 contained only cinnamic acid derivatives, F2 contained flavonoids and traces of cinnamic acid derivatives, and F3 contained hydrolysable tannins. Polymeric proanthocyanidins irreversibly bound the cartridge matrix and could not be recovered.

Table 4 shows the effects of F1, F2, and F3 treatments on *A*. *aegypti* larvae. The gut content elimination was detected 24 h after F1 incubation, while larval death was detected 24 h after F2 incubation. Three days after F1 or F2 incubations, the mortality rates were significantly higher (p < 0.05) than in the control. F1 killed 100% of the larvae 3 days after incubation, while F2 killed 66.7% of the larvae 8 days after incubation. No individual pupated after F2 incubation, while in the control individuals became adults. F3 incubation did not affect survival and development of larvae.

**Table 4:** *Aedes aegypti* L4 mortality rates after incubation with fractions (F1, F2, and F3) obtained after the separation of secondary metabolites from *Schinus terebinthifolius* leaf extract.

Parameter	Sample			
	F1	F2	F3	Control
**Mortality rate (%)**				
24 hours	0.0 a	10.0 ± 0.0 b	0.0 a	0.0 a
3 days	100.0 a	33.3 ± 5.7 b	6.7 ± 2.8 c	3.3 ± 2.8 c
8 days	100.0 a	66.7 ± 2.8 b	6.7 ± 2.8 c	3.3 ± 2.8 c
**Pupation (%)**	0.0 a	0.0 a	11.6 ± 5.7 b	10.0 ± 0.0 b
**Adult emergence (%)**	0.0 a	0.0 a	58.3 ± 5.7 b	68.3 ± 7.6 b
**Larvae that eliminated gut content (%)**	30.0 ± 5.0 a	0.0 b	0.0 b	0.0 b

F1: fraction eluted with Tris buffer, containing cinnamic acid derivatives. F2: fraction eluted with methanol, containing flavonoids and traces of cinnamic acid derivatives. F3: fraction eluted with methanol-acetic acid, containing hydrolysable tannins. Elimination of gut content was evaluated with 24 h of experiment. Pupation and adult emergence rates were calculated 8 days after larval incubation. Different letters at the same line indicate significant differences (p<0.05) between the treatments.

The lectin SteLL isolated from the leaf extract had a specific hemagglutinating activity of 27,334, and a purification fold of 337 compared to the extract. However, SteLL did not kill or affect the development of *A*. *aegypti* larvae at any tested concentration, and also did not induce elimination of the gut content by the larvae.

*S*. *terebinthifolius* leaf extract killed 100% of *A*. *salina* nauplii at all tested concentrations (0.125–1.0%), indicating that the leaf extract is potentially toxic to the environment at the concentrations that kill *A*. *aegypti* larvae. F1 and F2 at 1.0% (w/v) killed 73.3±5.8% and 13.3±5.8% of *A*. *salina* nauplii, respectively.

## Discussion

Integrated pest management has been considered an advance because it relies on a combination of common-sense practices and the most cost-effective methods for control of pests with the least possible hazard to people and the environment. The low cost and high efficacy make plant extracts an interesting possibility in integrated pest management programs [41]. Studies on the effects of essential oils from *S*. *terebinthifolius* fruits on mosquito larvae were performed with promising results, indicating the presence of larvicidal compounds in this plant [30], [42]. In this work, we evaluated a saline extract from *S*. *terebinthifolius* leaves for the presence of larvicidal agents with polar feature, and thus more advantageous than other insecticides with low solubility in aqueous systems.

After 12-h incubation, the *S*. *terebinthifolius* leaf extract induced acute reactions in *A*. *aegypti* larvae, being the most evident the elimination of the gut content and the darkening of the midgut. The elimination of the gut content enclosed in the peritrophic matrix indicated that the leaf extract interfered with food passage along the digestive tract, and suggests that the presence of the extract in the larval environment disturbed the structural organization of the midgut. Expelling gut contents has been reported as a defense mechanism of mosquito larvae aiming to excrete unabsorbed harmful compounds, such as DDT and some plant-derived insecticides [43], [44]. However, attempts of larvae to eliminate the harmful components of *S*. *terebinthifolius* leaf extract were not enough to prevent deleterious effects, since it chronically reduced larval survival, and interfered with development, even at concentrations lower than LC_50_. It is plausible that some of the toxic compounds from the extract remained inside the larvae midgut or may have been absorbed leading to mortality. In addition, there was a considerable loss of nutrients when the larvae expelled the gut content, which may also be linked to mortality.

Metamorphosis of the unfed individuals may have been prevented by food limitation in the experimental conditions since it has been shown that *A*. *aegypti* larvae will inhibit pupation if sufficient resources are unavailable [45]. Indeed, the pupation rate in the control was also very low. Therefore, in order to investigate if development was damaged mainly due to effects of the extract or to food restrictions, we performed the bioassays with the addition of food.

Treated fed larvae also eliminated the gut content after 24 h, but larval death was delayed in comparison with unfed larvae. This result may be ascribed to the availability of food resources that helped the larvae to compensate for part of the nutrient losses with the expulsion of the gut content. In spite of the delay of death in fed larvae, chronic disruption in development was evident, indicating that the extract was able to exert its deleterious effects even when food is provided. Thus, *S*. *terebinthifolius* leaf extract is clearly able to impair larval development even when there is no restriction in food availability.

The darkening of the midgut and hindgut, as well as of their contents, may have many reasons, and results from a number of mechanisms, including melanization due to activation of phenoloxidase cascade [46]. In turn, this activation can be due to several factors such as microbial infection, presence of components of cell walls from microorganisms and algae, parasitoids, action of proteolytic enzymes and tissue damage [47], [48], [49]. To verify if the darkening of the midgut of mosquito larvae incubated with the leaf extract was due to melanization, we added phenylthiourea (PTU, a strong phenoloxidase inhibitor) to the *S*. *terebinthifolius* leaf extract. The results showed that the strong pigmentation of larval midgut was still observed even in the presence of PTU, indicating that the midgut darkening was not related to melanization or resulted from other mechanisms, such as tissue injuries caused by the extract, or due to the accumulation of leaf extract into the larval midgut.

The absence of bacteria and yeasts in the leaf extract reveals that there were no microorganisms being introduced in the larval environment together with the extract, and thus there would be no elicitation of midgut melanization after incubation with the extract. In addition, the larval death could not be related to the presence of toxins released by microorganisms in the extract, but due to extract components solely.

Other plant-derived insecticides have shown deleterious effects in the midgut of mosquitoes, similar to the *S*. *terebinthifolius* leaf extract. For example, vacuolization and cell hypertrophy were caused by the ethanolic extract from *Magonia pubescens* bark [50], the methanolic extract from *Derris urucu* root [44], and an acetogenin from *Annona squamosa* [51] in the midgut of *A*. *aegypti*. Vacuolization, microvilli damage as well as cell lysis and death were reported as some of the effects of *Melia azedarach* extract on the midgut of *Culex quinquefasciatus* larvae [41]. The alkaloid pellitorine caused degeneration of epithelial cells at the anterior and posterior midgut regions of *A*. *aegypti* larvae, affecting the ability to osmoregulate, as well as damaged the gastric caeca, including cells important in ion transport [52].

The damage to digestive cells in the midgut of *A*. *aegypti* larvae caused by the *S*. *terebinthifolius* leaf extract may have impaired digestive and absorption processes in the larval midgut, compromising survival, and disrupting larval mosquito development. The regenerative cells play an important role in development since they start their division in the last larval instar and finish in the early pupal stage, resulting in renewing of intestinal epithelium, an essential step in metamorphosis [53]. Once regenerative cell division was reduced in treated larvae, it prevented transformation of the midgut, and organ remodeling during pupation.

Damage to enteroendocrine cells have the potential to disrupt homeostasis throughout the larval body, since these cells have been reported to release peptides, monoamines, and other substances that supposedly have paracrine effects on regenerative cells and enzyme production by digestive cells [54].

Results from TUNEL assay revealed that components of *S*. *terebinthifolius* leaf extrac caused cleavage of DNA in the midgut cells. This can result from activation of apoptotic, necrotic, and autolysis processes or severe DNA damage caused by the extract [55].

In summary, the *S*. *terebinthifolius* leaf extract promoted larval mortality probably due to: 1) induction of acute reaction by larvae, which expelled the gut contents resulting in lost food nutrients; 2) blocking of digestion and absorption of nutrients due to cytotoxic effects on digestive cells and disruption of microvilli; 3) deregulation of digestion and secretion of substances either in the gut lumen or in the hemolymph due to impairment of enteroendocrine cells; and 4) disruption of gut homeostasis due to the partial detachment of the peritrophic matrix and extensive tissue disorganization in the midgut.

Secondary metabolites from leaf extract of *S*. *terebinthifolius* were semi-purified in order to check the deleterious effects of each class of secondary metabolites in *A*. *aegypti* larvae. The fraction containing cinnamic acid derivatives (F1) was able to kill all larvae in 3 days, while the fraction containing mainly flavonoids (F2) promoted high mortality after 8 days. These results indicate that the cinnamic acid derivates are the main responsible for the lethal effect, but the flavonoids are also larvicidal components of the extract.

Cinnamic acid derivatives and aglycone flavonoids have been previously reported to have insecticidal effects. Aglycone flavonoids, such as quercetin and kaempferol, were detected in larvicidal extracts from *Moringa oleifera* flowers [9] and *Gardenia ternifolia* aerial parts [56], and these flavonoids, when isolated, were able to inhibit the cytochrome P-450 dependent ecdysone 20-monooxygenase activity in *A*. *aegypti* [57]. Quercetin also negatively affected weight gain of *Bombyx mori* larvae, as well as induced detoxifying enzyme activity [58]. The toxicity of methanolic *D*. *urucu* root extract, which induced larvae to excrete feces and caused midgut damage, has been attributed to an isoflavonoid called rotenone [44]. The cinnamic acid derivatives, cinnamaldehyde and cinnamyl acetate, showed strong larvicidal properties against fourth-instar larvae of *A*. *aegypti* [59].

When mosquito larvae were incubated with F1, the gut content was also eliminated, suggesting the involvement of cinnamic acid derivatives in this phenomenon. However, the number of larvae with this phenotype was lower than in the set of larvae treated with the complete extract, indicating that not only cinnamic acid derivatives induce the elimination of the gut content, but also other extract components. The elimination of the gut content is probably a response to an overload of foreign and potentially toxic compounds in the gut lumen, as discussed above. Indeed, some compounds that are low active or inactive when isolated may cause strong effects when in combination with other active principles [60].

The leaf extract used here was prepared using the same procedure described by Gomes, et al. [28] for obtaining crude extract from which the lectin SteLL was isolated. Since lectins have been reported as larvicidal agents against *A*. *aegypti* [34], [61], [62], [63], we hypothesized that SteLL could be involved in the deleterious effects of *S*. *terebinthifolius* leaf extract on larvae. However, SteLL did not kill the larvae or induce gut content elimination, which suggests that it is not a component of the larvicidal property of the extract, or is not effective when separated of other extract components. This is reinforced by the very high purification fold of the sample tested, which indicates that lectin molecules were more concentrated in SteLL than in the extract, and thus, larvae should be affected if the lectin was involved in the deleterious effects of *S*. *terebinthifolius* leaf extract.

Plant compounds are usually less hazardous to the environment than synthetic insecticides, but this does not exclude the possibility that a natural insecticide could be harmful to non-target organisms. Thus, we evaluated the environmental toxicity of *S*. *terebinthifolius* leaf extract by determining its potential in promoting death of *A*. *salina* nauplii. The extract and F1 were toxic to *A*. *salina* nauplii, while F2 showed low toxicity n this species. These findings revealed that although the cinnamic acid derivatives have had a faster larvicidal effect than the flavonoids, these last would be a better alternative from an environmental standpoint. The extract and preparations containing the cinnamic acid derivatives can be effectively used, for example, in flowerpot plates, gully traps, the collar of toilet bowls, roof gutters, scupper drains, air-conditioner trays, old tires, and bottles, among others. However, use in domestic containers for storage of water intended for human and animal consumption, such as water tanks, aquariums, and fountains should not currently be considered safe.

## Conclusions

*S*. *terebinthifolius* leaf extract showed larvicidal activity on *A*. *aegypti* as well as interfered with development from pupal to adult stages. These effects are linked to damage of digestive, enteroendocrine, and regenerative cells in midgut of the larvae, as well as induction of structural disorganization of the gut and elimination of the gut content. Caution is required in the use of the extract as a larvicide against *A*. *aegypti* due to the toxic effects detected in the *A*. *salina* assay. Cinnamic acid derivatives and flavonoids are responsible for larvicidal effect of the extract. The cinnamic acid derivatives killed individuals in a shorter time but were toxic to *A*. *salina*, while the flavonoids, although effective in a longer period, were exempt from toxicity to this microcrustacean. On the other hand, the lectin (SteLL) seems not to be involved in the *A*. *aegypti* larvae damage.

## References

[1] Bhatt S, Gething PW, Brady OJ, Messina JP, Farlow AW, Moyes CL, et al The global distribution and burden of dengue. Nature. 2013;496: 504–507. 10.1038/nature12060 23563266 PMC3651993

[2] World Health Organization (2014) Dengue and severe dengue. Fact sheet N° 117.

[3] Maciel-de-Freitas R, Avendanho FC, Santos R, Sylvestre G, Araújo SC, Lima JBP, et al Undesirable consequences of insecticide resistance following *Aedes aegypti* control activities due to a dengue outbreak. PLoS ONE. 2014;9: e92424 10.1371/journal.pone.0092424 24676277 PMC3968006

[4] Aiub CAF, Coelho ECA, Sodré E, Pinto LFR, Felzenszwalb I. Genotoxic evaluation of the organophosphorous pesticide temephos. Genet Mol Res. 2002;1: 159–166. 14963843

[5] Melo-Santos MAV, Varjal-Melo JJM, Araújo AP, Gomes TCS, Paiva MHS, Regis LN, et al Resistance to the organophosphate temephos: mechanisms, evolution and reversion in an *Aedes aegypti* laboratory strain from Brazil. Acta Trop. 2010;113: 180–189. 10.1016/j.actatropica.2009.10.015 19879849

[6] Ocampo C, Salazar-Terreros M, Mina N, Mcallister J, Brogdon W. Insecticide resistance status of *Aedes aegypti* in 10 localities of Colombia. Acta Trop. 2011;118: 37–44. 10.1016/j.actatropica.2011.01.007 21300017

[7] Polson KA, Brogdon WG, Rawlins SC, Chadee DD. Characterization of insecticide resistance in Trinidadian strains of *Aedes aegypti* mosquitoes. Acta Trop. 2011;117: 31–38. 10.1016/j.actatropica.2010.09.005 20858454

[8] Vontas J, Kioulos E, Pavlidi N, Morou E, della Torre A, Ranson H. Insecticide resistance in the major dengue vectors *Aedes albopictus* and *Aedes aegypti* . Pest Biochem Physiol. 2012;104: 126–131.

[9] Pontual EV, Napoleão TH, Assis CRD, Bezerra RS, Xavier HS, Navarro DMAF, et al Effect of *Moringa oleifera* flower extract on larval trypsin and acethylcholinesterase activities in *Aedes aegypti* . Arch Insect Biochem Physiol. 2012;79: 135–152. 10.1002/arch.21012 22392801

[10] Murray T, Miles C, Daniels C. Natural insecticides. Washington State University, Oregon State University, University of Idaho PNW649; 2013.

[11] Amer A, Mehlhorn H. Larvicidal effects of various essential oils against *Aedes*, *Anopheles*, and *Culex* larvae (Diptera, Culicidae). Parasitol Res. 2006;99: 466–472. 16642386 10.1007/s00436-006-0182-3

[12] Costa MS, Pinheiro DO, Serrão JE, Pereira MJB. Morphological changes in the midgut of *Aedes aegypti* L. (Diptera: Culicidae) larvae following exposure to an *Annona coriacea* (Magnoliales: Annonaceae) extract. Neotrop Entomol. 2012;41: 311–314. 10.1007/s13744-012-0050-z 23950067

[13] Deletre E, Martin T, Campagne P, Bourguet D, Cadin A, Menut C, et al Repellent, irritant and toxic effects of 20 plant extracts on adults of the malaria vector *Anopheles gambiae* mosquito. PLoS ONE. 2013;8: e82103 10.1371/journal.pone.0082103 24376515 PMC3871167

[14] Kamiabi F, Jaal Z, Keng CL. Bioefficacy of crude extract of *Cyperus aromaticus* (Family: Cyperaceae) cultured cells, against *Aedes aegypti* and *Aedes albopictus* mosquitoes. Asian Pac J Trop Biomedic. 2013;3: 767–775. 10.1016/S2221-1691(13)60153-7 24075340 PMC3761134

[15] Navarro DMAF, Oliveira PES, Potting RJP, Brito AC, Fital SJF, Sant’Ana AEG. The potential attractant or repellent effects of different water types on oviposition in *Aedes aegypti* L. (Dipt., Culicidae). J Appl Entomol. 2003;127: 46–50.

[16] Pontual EV, Santos NDL, Moura MC, Coelho LCBB, Navarro DMAF, Napoleão TH, et al Trypsin inhibitor from *Moringa oleifera* flowers interferes with survival and development of *Aedes aegypti* larvae and kills bacteria inhabitant of larvae midgut. Parasitol Res. 2014;113: 727–733. 10.1007/s00436-013-3702-y 24271154

[17] Paiva PMG, Pontual EV, Napoleão TH, Coelho LCBB. Lectins and trypsin inhibitors from plants: Biochemical characteristics and adverse effects on insect larvae. New York: Nova Science Publishers, Inc.; 2013.

[18] Brusca GJ, Brusca RC. Invertebrates. Sunderland: Sinauer Associates; 2003.

[19] Ray K, Mercedes M, Chan D, Choi CY, Nishiura JT. Growth and differentiation of the larval mosquito midgut. J Insect Sci. 2007;9: 1–13. 10.1673/031.009.5501 PMC3011905 20053117

[20] Fernandes KM, Neves CA, Serrão JE, Martins GF. *Aedes aegypti* midgut remodeling during metamorphosis. Parasitol Int. 2014;63: 506–512. 10.1016/j.parint.2014.01.004 24472855

[21] Lorenzi H. Árvores brasileiras: manual de identificação e cultivo de plantas arbóreas do Brasil. Nova Odessa: Plantarum; 2008;

[22] Balbachas A. As plantas curam. São Paulo: Missionária; 1959.

[23] Lindenmaier DS. Etnobotânica em comunidades indígenas Guaranis no Rio Grande do Sul. Santa Cruz do Sul: Universidade de Santa Cruz do Sul; 2008.

[24] Jain MK, Yu BZ, Rogers JM, Smith AE, Boger ETA, Ostrander RL, et al Specific competitive inhibitor of secreted phospholipase A2 from berries of *Schinus terebinthifolius* . Phytochemistry. 1995;39: 537–547. 7576451 10.1016/0031-9422(94)00960-2

[25] Matsuo AL, Figueiredo CR, Arruda DC, Pereira FV, Scutti JAB, Massaoka MH, et al α-Pinene isolated from *Schinus terebinthifolius* Raddi (Anacardiaceae) induces apoptosis and confers antimetastatic protection in a melanoma model. Biochem Biophys Res Commun. 2011;411: 449–454. 10.1016/j.bbrc.2011.06.176 21756878

[26] Pawlowski Â, Kaltchuk-Santos E, Zini CA, Caramão EB, Soares GLG. Essential oils of *Schinus terebinthifolius* and *S*. *molle* (Anacardiaceae): Mitodepressive and aneugenic inducers in onion and lettuce root meristems. South Afric J Bot. 2012;80: 96–103.

[27] Cavalher-Machado SC, Rosas EC, Brito FA, Heringe AP, Oliveira RR, Kaplan MAC, et al The anti-allergic activity of the acetate fraction of *Schinus terebinthifolius* leaves in IgE induced mice paw edema and pleurisy. Int Immunopharmacol. 2008;8: 1552–1560. 10.1016/j.intimp.2008.06.012 18672096

[28] Gomes FS, Procópio TF, Napoleão TH, Coelho LCBB, Paiva PMG. Antimicrobial lectin from *Schinus terebinthifolius* leaf. J Appl Microbiol. 2013;114: 672–679. 10.1111/jam.12086 23190078

[29] Coelho AAM, de Paula JE, Espíndola LS. Atividade larvicida de extratos vegetais sobre *Aedes aegypti* (L.) (Diptera: Culicidae), em condições de laboratório. BioAssay. 2009;4: 3.

[30] Silva AG, Almeida DL, Ronchi SN, Bento AC, Scherer R, Ramos AC, et al The essential oil of Brazilian pepper, *Schinus terebinthifolia* Raddi in larval control of *Stegomyia aegypti* (Linnaeus, 1762). Parasit Vectors. 2010;3: 79 10.1186/1756-3305-3-79 20799936 PMC2936394

[31] Roberts EH, Cartwright RA, Wood DJ. The flavonols of tea. J Sci Food Agric. 1956;7: 637–646.

[32] Wagner H, Bladt S. Plant drug analysis. New York: Springer; 1996.

[33] Harborne JB. Phytochemical Methods: a guide to modern techniques of plant analysis. London: Chapman & Hall; 1998.

[34] Napoleão TH, Pontual EV, Lima TA, Santos NDL, Sá RA, Coelho LCBB, et al Effect of *Myracrodruon urundeuva* leaf lectin on survival and digestive enzymes of *Aedes aegypti* larvae. Parasitol Res. 2012;110: 609–616. 10.1007/s00436-011-2529-7 21735148

[35] Lowry OH, Rosebrough NJ, Farr AL, Randall RJ. Protein measurement with the Folin phenol reagent. J Biol Chem. 1951;193: 265–275. 14907713

[36] Navarro DMAF, Silva PCB, Silva MR, Napoleão TH, Paiva PMG. Larvicidal activity of plant and algae extracts, essential oils and isolated chemical constituents against *Aedes aegypti* . Nat Prod J. 2013;3: 268–291.

[37] World Health Organization. Instructions for determining the susceptibility or resistance of mosquito larvae to insecticides. WHO/VBC/81.807. p 1–6; 1981.

[38] Fernandes KM, Araújo VA, Serrão JE, Martins GF, Campos LAO, Neves CA. Quantitative Analyses of the digestive and regenerative cells of the midgut of *Melipona quadrifasciata anthidioides* (Hymenoptera; Apidae). Sociobiology. 2010;56: 489–505.

[39] Preuss U, Landsberg G, Scheidtmann KH. Novel mitosis-specific phosphorylation of histone H3 at Thr11 mediated by Dlk/ZIP kinase. Nucleic Acids Res. 2003;31: 878–885. 12560483 10.1093/nar/gkg176 PMC149197

[40] Meyer BN, Ferrigini NR, Putman JE, Jacobson LB, Nichols DE, McLaughlin JL. Brine shrimp: a convenient general bioassay for active plant constituents. Planta Med. 1982;45: 31–34. 7100305

[41] Al-Mehmadi RM, Al-Khalaf AA. Larvicidal and histological effects of *Melia azedarach* extract on *Culex quinquefasciatus* Say larvae (Diptera: Culicidae). J King Saud Univ. 2010;22: 77–85.

[42] Pratti DLA, Ramos AC, Scherer R, Cruz ZMA, Ary G. Mechanistic basis for morphological damage induced by essential oil from Brazilian pepper tree, *Schinus terebinthifolia*, on larvae of *Stegomyia aegypti*, the dengue vector. Parasit Vectors. 2015;8: 136 10.1186/s13071-015-0746-0 25886180 PMC4349733

[43] Abedi ZH, Brown AWA. Peritrophic membrane as vehicle for DDT and DDE excretion in *Aedes aegypti* larvae. Ann Entomol Soc America. 1961;54: 539–542.

[44] Gusmão DS, Páscoa V, Mathias L, Vieira IJC, Braz-Filho R, Lemos FJA. *Derris* (*Lonchocarpus*) *urucu* (Leguminosae) extract modifies the peritrophic matrix structure of *Aedes aegypti* (Diptera:Culicidae). Mem Inst Oswaldo Cruz. 2002;97: 371–375. 12051197 10.1590/s0074-02762002000300017

[45] Levi T, Ben-Dov E, Shahi P, Borovsky D, Zaritsky A. Growth and development of *Aedes aegypti* larvae at limiting food concentrations. Acta Trop. 2014;133: 42–44. 10.1016/j.actatropica.2014.02.001 24524949

[46] Shao Q, Yang B, Xu Q, Li X, Lu Z, Wang C, et al Hindgut innate immunity and regulation of fecal microbiota through melanization in insects. J Biol Chem. 2012;287: 14270–14279. 10.1074/jbc.M112.354548 22375003 PMC3340165

[47] Ashida M, Ishizaki Y, Iwahana H. Activation of pro-phenoloxidase by bacterial cell wall or β-1,3-glucans in plasma of the silkworm, *Bombyx mori* . Biochem Biophys Res Commun. 1983;113: 562–564. 6409105 10.1016/0006-291x(83)91762-x

[48] Rowley AF; Brookman JL, Ratcliffe NA. Possible involvement of the prophenoloxidase system of the locust, *Locusta migratoria*, in antimicrobial activity. J Inv Pathol. 1990;56: 31–38.

[49] Silva CCA. Aspectos do sistema imunológico dos insetos. Biotecnol Ciên Desenv. 2002;24: 68–72.

[50] Arruda W, Oliveira GMC, Silva IG. Toxicidade do extrato etanólico de *Magonia pubescens* sobre larvas de *Aedes aegypti* . Rev Soc Bras Med Trop. 2003;36: 17–25. 12715059 10.1590/s0037-86822003000100004

[51] Costa MS, Cossolin JFS, Pereira MJB, Sant'Ana AEG, Lima MD, Zanuncio JC, et al Larvicidal and cytotoxic potential of squamocin on the midgut of *Aedes aegypti* (Diptera: Culicidae). Toxins. 2014;6: 1169–1176. 10.3390/toxins6041169 24674934 PMC4014726

[52] Perumalsamy H, Kim J-R, Oh SM, Jung JW, Ahn Y-J, Kwon HW. Novel histopathological and molecular effects of natural compound pellitorine on larval midgut epithelium and anal gills of *Aedes aegypti* . PLoS ONE. 2013;8: e80226 10.1371/journal.pone.0080226 24260359 PMC3832413

[53] Nishiura JT, Ho P, Ray K. Methoprene interferes with mosquito midgut remodeling during metamorphosis. J Med Entomol. 2003;40: 498–507. 14680117 10.1603/0022-2585-40.4.498

[54] Brown MR, Raikhel AS, Lea AO. Ultrastructure of midgut endocrine cells in the adult mosquito, *Aedes aegypti* . Tissue Cell. 1985;17: 709–721. 4060146 10.1016/0040-8166(85)90006-0

[55] Fink SL, Cookson BT. Apoptosis, pyroptosis, and necrosis: Mechanistic description of dead and dying eukaryotic cells. Infect Immun. 2005;73: 1907–1916. 15784530 10.1128/IAI.73.4.1907-1916.2005 PMC1087413

[56] Ochieng CO, Midiwo JO, Owuor PO. Anti-plasmodial and larvicidal effects of surface exudates of *Gardenia ternifolia* aerial parts. Res J Pharmacol. 2010;4: 45–50.

[57] Mitchell MJ, Keogh DP, Crooks JR, Smith SL. Effects of plant flavonoids and other allelochemicals on insect cytochrome P-450 dependent steroid hydroxylase activity. Insect Biochem Mol Biol. 1993;23: 65–71. 8485518 10.1016/0965-1748(93)90083-5

[58] Zhang YE, Ma HJ, Feng DD, Lai XF, Chen ZM, Xu MY, et al Induction of detoxification enzymes by quercetin in the silkworm. J Econ Entomol. 2012;105: 1034–1042. 22812145 10.1603/ec11287

[59] Cheng SS, Liu JY, Tsai KH, Chen WJ, Chang ST. Chemical composition and mosquito larvicidal activity of essential oils from leaves of different *Cinnamomum osmophloeum* provenances. J Agric Food Chem. 2004;14: 4935–4400. 10.1021/jf0497152 15237942

[60] Shaalan EA, Canyon D, Younes MWF, Abdel-Wahab M, Mansour A-H. A review of botanical phytochemicals with mosquitocidal potential. Environ Int. 2005;31: 1149–1166. 15964629 10.1016/j.envint.2005.03.003

[61] Coelho JS, Santos NDL, Napoleão TH, Gomes FS, Ferreira RS, Zingali RB, et al Effect of *Moringa oleifera* lectin on development and mortality of *Aedes aegypti* larvae. Chemosphere. 2009;77: 934–938. 10.1016/j.chemosphere.2009.08.022 19747711

[62] Sá RA, Santos NDL, Silva CSB, Napoleão TH, Gomes FS, Cavada BS, et al Larvicidal activity of lectins from *Myracrodruon urundeuva* on *Aedes aegypti*. Comp. Biochem. Physiol C. 2009;149: 300–306. 10.1016/j.cbpc.2008.08.004 18761426

[63] Agra-Neto AC, Napoleão TH, Pontual EV, Santos NDL, Luz LA, Oliveira CMF, et al Effect of *Moringa oleifera* lectins on survival and enzyme activities of *Aedes aegypti* larvae susceptible and resistant to organophosphate. Parasitol Res. 2014;113: 175–184. 10.1007/s00436-013-3640-8 24142287

